# The Inhibition of Antibiotic Production in *Streptomyces coelicolor* Over-Expressing the TetR Regulator SCO3201 IS Correlated With Changes in the Lipidome of the Strain

**DOI:** 10.3389/fmicb.2020.01399

**Published:** 2020-06-23

**Authors:** Jun Zhang, Qiting Liang, Zhongheng Xu, Miao Cui, Qizhong Zhang, Sonia Abreu, Michelle David, Clara Lejeune, Pierre Chaminade, Marie-Joelle Virolle, Delin Xu

**Affiliations:** ^1^Key Laboratory of Eutrophication and Red Tide Prevention of Guangdong Higher Education Institutes, Department of Ecology, School of Life Sciences and Technology, Engineering Research Center of Tropical and Subtropical Aquatic Ecological Engineering, Ministry of Education, Institute of Hydrobiology, Jinan University, Guangzhou, China; ^2^Université Paris-Saclay, Lipides, Systèmes Analytiques et Biologiques, Châtenay-Malabry, France; ^3^Group “Energetic Metabolism of Streptomyces”, Institute for Integrative Biology of the Cell (I2BC), CEA, CNRS, Univ. Paris-Sud, INRA, University Paris-Saclay, Gif-sur-Yvette, France

**Keywords:** *Streptomyces coelicolor*, antibiotics, TetR regulator, fatty acid metabolism, RNA-seq

## Abstract

In condition of over-expression, SCO3201, a regulator of the TetR family was previously shown to strongly inhibit antibiotic production and morphological differentiation in *Streptomyces coelicolor* M145. In order to elucidate the molecular processes underlying this interesting, but poorly understood phenomenon, a comparative analysis of the lipidomes and transcriptomes of the strain over-expressing *sco3201* and of the control strain containing the empty plasmid, was carried out. This study revealed that the strain over-expressing *sco3201* had a higher triacylglycerol content and a lower phospholipids content than the control strain. This was correlated with up- and down- regulation of some genes involved in fatty acids biosynthesis (*fab*) and degradation (*fad*) respectively, indicating a direct or indirect control of the expression of these genes by SCO3201. In some instances, indirect control might involve TetR regulators, whose encoding genes present in close vicinity of genes involved in lipid metabolism, were shown to be differentially expressed in the two strains. Direct interaction of purified His_6_-SCO3201 with the promoter regions of four of such TetR regulators encoding genes (*sco0116*, *sco0430*, *sco4167*, and *sco6792*) was demonstrated. Furthermore, *fasR* (*sco2386*), encoding the activator of the main fatty acid biosynthetic operon, *sco2386*-*sco2390*, has been shown to be an illegitimate positive regulatory target of SCO3201. Altogether our data demonstrated that the *sco3201* over-expressing strain accumulates TAG and suggested that degradation of fatty acids was reduced in this strain. This is expected to result into a reduced acetyl-CoA availability that would impair antibiotic biosynthesis either directly or indirectly.

## Introduction

*Streptomyces* are Gram-positive filamentous bacteria living in terrestrial, marine and fresh water ecosystems where they play essential biological roles (van der Meij et al., 2017). These bacteria have great industrial and economic importance since they produce most of the antibiotics in current use and a variety of other bioactive molecules useful to human health (e.g., anti-cancer and anti-inflammatory drugs), agriculture (e.g., fungicides, pesticides, insecticides, and herbicides), or industry (relevant enzymes) (Worthen, 2008; Barka et al., 2016; Baral et al., 2018). On solid medium, *Streptomyces* are characterized by a complex differentiation cycle that starts with the germination of a spore (Bobek et al., 2017), giving rise to a vegetative mycelium. However, when the bacteria faces conditions of nutritional stress, a programmed cell death (PCD) process is triggered (Flardh and Buttner, 2009; Manteca et al., 2010), resulting into the partial lysis of the vegetative mycelium that provides nutrients to support the development of an aerial mycelium (Mendez et al., 1985; Granozzi et al., 1990; Chater, 2006). Subsequently, the ends of the aerial hyphae tips differentiate into unigenomic spores that will disseminate in the environment. In solid-grown cultures, the production of secondary metabolites usually coincides with the onset of morphological differentiation indicating the existence of regulatory features common to these two processes (Chater, 2006; Liu et al., 2013; Wei et al., 2018). Regulatory cascades triggered by the sensing of extracellular and/or intracellular signals are involved in the control of the developmental program (Pereira et al., 2011). These include Ser/Thr/Tyr eukaryotic-like protein kinases or phosphatases (Petrickova and Petricek, 2003; Pereira et al., 2011), membrane-anchored sensory Histidine kinases from two-component systems (Rodriguez et al., 2013), as well as transcriptional regulators able to interact with specific ligands (Romero-Rodriguez et al., 2015). The latter class include regulators of the TetR family that bear a highly conserved helix-turn-helix (HTH) motif usually located in the N-terminal part of the protein involved in DNA binding, and a much more variable ligand-binding domain (Yu et al., 2010; Cuthbertson and Nodwell, 2013). TetR regulators act most often as transcriptional repressors since their binding site usually overlaps the promoter region of their target genes. However when their binding site is located upstream of the −35 promoter region of their regulatory targets, they can also act as activators (Cuthbertson and Nodwell, 2013). The behavior of the couple TetR/regulatory target is intrinsically complex and relies on the intracellular abundance of the specific ligand of the TetR regulator that usually hinders the interaction of the TetR with its target sites (Ahn et al., 2007). We have previously identified a TetR-like regulator SCO3201, whose over-expression in *S. coelicolor* caused inhibition of the biosynthesis of the blue polyketide antibiotic, actinorhodin (ACT) as well as of morphological differentiation of this strain (Xu et al., 2010). SCO3201 was proposed to govern the expression of genes encoding TetR-like regulators and/or of their regulatory targets but only in condition of over-expression since its disruption had no obvious phenotype (Xu et al., 2010). The ability of SCO3201 to control the expression of numerous “illegitimate” targets was attributed to the presence of a long N terminal extension, revealed by crystallographic studies, that would stabilize the interaction of SCO3201 with weakly similar binding sites (Xu et al., 2014). In this study, we performed a comparative RNA-Seq analysis of *S. coelicolor* carrying the empty vector (M145/pWHM3) or the *sco3201* over-expression construct (M145/pWHM3-*sco3201*). This study revealed that the expression of several pathways was affected by the over-expression of *sco3201*. Since a putative negative correlation between antibiotics biosynthesis and TAG content was previously reported (Le Marechal et al., 2013; Esnault et al., 2017), we mainly examined the consequences of *sco3201* over-expression on fatty acid metabolism.

## Materials and Methods

### *Streptomyces* Strains and Culture Media

The wild-type strain of *S. coelicolor* M145 (Bentley et al., 2002) containing the empty high-copy-number plasmid pWHM3 (Vara et al., 1989) and the previously constructed strain overexpressing *sco3201* (*S. coelicolor* M145/pWHM3-*sco3201*) were used in this study (Xu et al., 2010). R2YE solid medium was used to cultivate *Streptomyces* strains. Thiostrepton (50 μg/ml) was added to the cultures when necessary.

### Lipid Extraction and Analysis by LC/Corona-CAD and LC/MS

Lipid extraction was performed from four independent cultures of *S. coelicolor* M145/pWHM3 and *S. coelicolor* M145/pWHM3-*sco3201* using a procedure derived from the Folch’s method (Folch et al., 1957). A defined volume (4.5 ml) of chloroform:methanol (1:2) was added to 10 mg of lyophilized *Streptomyces* mycelium and vortexed during 30 s. The mixture was left at ambient temperature for 1 h, then 1.25 ml of water was added. The mixture was vortexed 30 s then centrifuged (1000 g/10 min) to obtain phase separation. The lower organic phase was collected and the upper aqueous phase was submitted to a second extraction by adding 2 ml of chloroform: methanol (85:15). The two organic phases were pooled and evaporated under a stream of nitrogen at room temperature. The dry residue was dissolved in 400 μl of isooctane: chloroform (4:1) before analysis. The chromatographic conditions were as described previously (Folch et al., 1957). Briefly, lipid class analysis was performed with an Inertsil Silica (150 mm × 2.1 mm I.D, 5μm) column (GL Sciences Inc., Tokyo, Japan) thermostated at 40°C. The HPLC instrumentation consisted of system Dionex U-3000 RSLC (Thermo Fisher, Villebon, France). A quaternary solvent gradient (Supplementary Table 2) was used to elute the whole lipid classes present in the sample by increasing order of polarity. Lipid classes identification was verified by coupling the chromatographic separation to mass spectrometry. MS analyses were performed with a LTQ-Orbitrap Velos Pro (Thermo Fisher Scientific) equipped with an APPI ion source. The MS^2^ and MS^3^ spectra were obtained in data dependent acquisition (DDA) mode. Lipid detection was performed using a Corona-CAD system (ESA, Chelmsford, MA, United States) (Abreu et al., 2017); the signal was acquired with a Chromeleon data station (Thermo Fisher Scientific, Villebon-sur-Yvette, France). Corona-CAD is an universal detector used in liquid chromatography and described by Dixon and Peterson (2002). The differences in composition of the lipid classes in the samples are expressed as peak areas. All data were subjected to the Student’s *t*-test using R 3.3.2 and the “multcompView” package. The results obtained are presented as the mean ± standard deviation, a *p* < 0.05 was considered as statistically significant.

### RNA Isolation, Transcriptome Analysis by RNA-Seq and qRT-PCR

10^7^ spores of each strain were plated on cellophane discs laid on the surface of R2YE solid medium and incubated at 28°C for 24, 36, or 48 h. Mycelium was collected from three independent R2YE plates at various time points and total RNA was isolated using TRIzol-based reagent (Life Technologies, CA, United States) following the manufacturer’s instructions. RNA integrity was confirmed by agarose gel electrophoresis, and the absence of genomic DNA was checked by Agilent 2100 RNA 6000 Nano kit. Ribosomal RNA were removed with a Ribo-Zero Magnetic Gold Kit (Epicentre Biotechnologies, Madison, WI, United States). Samples were sent to BaseClear, an independent and accredited service laboratory for DNA-based research to carry out transcriptome analysis by RNA-Seq. The sequences obtained on an Illumina sequencer were filtered for non-coding RNAs and analyzed with CLCbio bioinformatics software packages using the annotated *S. coelicolor* genome as reference (Bentley et al., 2002). Expression values were shown as reads/kb of exon model/million mapped reads, i.e., dividing the total number of exon reads (in this case one exon per reference sequence) by the number of mapped reads (in millions) times the exon length (in this case the length of the reference sequence).

All the differentially expressed genes of interest that were identified by RNA-Seq were subsequently validated by reverse transcription-PCR (qRT-PCR). For qRT-PCR, mtwo micrograms of each RNA samples was used as a template for cDNA synthesis with random primers, using the MLV Reverse Transcriptase (catalog no. M1705; Promega). The program for cDNA synthesis was 70°C/5 min, 42°C/60 min followed by 10 min at 72°C. Ten percent of the cDNA synthesis reaction mixture (2 μl) was used as a template for each subsequent PCR, using the gene-specific primer pairs listed in Supplementary Table 1. The PCR program used was as follows: (i) 95°C/1 min, (ii) 95°C/20 s (iii) 62°C/20 s, and (iv) 72°C/20 s with the second to the fourth steps repeated for 40 cycles. Negative controls were carried out without the addition of reverse transcriptase in order to demonstrate that amplified products were derived from RNA transcripts and not from chromosomal DNA.

The RNA seq dataset reported in the present study was deposited in the NCBI databases with accession number of PRJNA624242.

### Electrophoretic Mobility Shift Assay (EMSA)

The promoter regions of *sco0116*, *sco0430*, *sco2386*, *sco2390*, *sco4167*, and *sco6792* were amplified from *S. coelicolor* M145 genomic DNA by PCR using primer pairs 0116F/0116R, 0430F/0430R, 2386F/2386R, 2390F/2390R, 4167F/4167R, and 6792F/6792R (Supplementary Table 1), respectively. The amplified PCR products were 5′ labeled with FITC (Fluoresceine IsoThioCyanate) by PCR using primer Plabel (Supplementary Table 1). Six-histidine-tagged SCO3201 (His_6_-SCO3201) was purified from *E. coli* as previously described (Xu et al., 2010). EMSA was performed as described previously (Xu et al., 2010). Briefly, 0.522 pmol of each of the promoter regions was incubated with purified His_6_-SCO3201 in various concentrations for 15 min at room temperature in binding buffer (10 mM Tris-HCl, 50 mM KCl, 1 mM DTT, pH 7.5) in a total volume of 20 μl. In order to assess binding specificity, competition assays were carried out using excess amounts of specific competitors of unlabeled *sco3201* promoter region or non-specific competitors of an unlabeled unrelated DNA probe, respectively. After incubation, the reaction mixtures were loaded on a 5% native polyacrylamide gel pre-run at 100 V for 30 min and run at 100 V for 90 min in running buffer (45 mM Tris-HCl, pH 8.3, 45 mM boric acid, 10 mM EDTA). The DNA signal was visualized by fluorescence imaging using a Bio-rad ChemiDoc MP (United States).

## Results

### The Over-Expression of *sco3201* Alters the Lipidome of *S. coelicolor*

**FIGURE 1:**
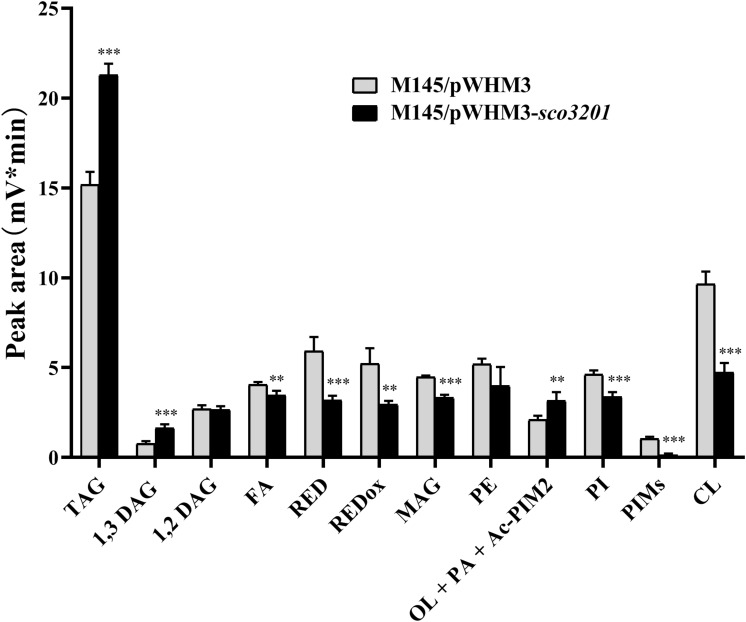
Analysis of total lipid content of *S. coelicolor* M145/pWHM3 (black histograms) and *S. coelicolor* M145/pWHM3-*sco3201* (light gray histograms) by LC/MS expressed in arbitrary unit (mV*min) representing the peak area of each lipid specie detected by the Corona-CAD system used in this study. TAG stands for TriAcylGlycerol, DAG for DiAcylGlycerol, FA for Fatty Acids, RED for Undecylprodigiosin, REDox for oxidized Undecylprodigiosin, MAG for MonoAcylGlycerol, PE for PhosphatidylEthanolamine, OL for Ornithine Lipid, PA for Phosphatidic Acid, Ac-PIM2 for monoACyl PhosphatidylInositol diMannoside, PI for Phosphatidyl Inositol, PIMs for PhosphatidylInositol Mannosides and CL for CardioLipid. Significant difference of lipid content between the two strains are represented by an asterisk (ANOVA, ^∗∗∗^*p* < 0.001; ^∗∗^*p* < 0.01).

Since our previous results indicated the existence of a negative correlation between TAG content and antibiotic biosynthesis (Le Marechal et al., 2013; Esnault et al., 2017), analysis of the lipid content of M145/pWHM3 and M145/pWHM3-*sco3201* was carried out using LC/Corona-CAD (Abreu et al., 2017). This study revealed that the content in storage lipids of the triacylglycerol family (TAG) and of its direct precursor 1,3 diacylglycerol (DAG) were respectively 1.3 and 1.8-fold more abundant in M145/pWHM3-*sco3201* than in M145/pWHM3 (Figure 1 and Supplementary Figure 1). The mixture Ornithine Lipid (OL)/Phosphatidic Acid (PA)/Monoacyl phosphatidylinositol dimannoside (Ac-PIM2) was also 1.3-fold more abundant in M145/pWHM3-*sco3201* than in M145/pWHM3. *S. coelicolor* is known to have a very low content in OL (Le Marechal et al., 2013; Esnault et al., 2017) and this study revealed that it has also a low PhosphatidylInositol Mannosides (PIM) and thus likely a low Ac-PIM2 content (Figure 1 and Supplementary Figure 1). This suggested that the increase in the intensity of the OL/PA/Ac-PIM2 peak was likely due to an increase of its PA content, another direct precursor of TAG. Consistently, biosynthetic intermediates such as free fatty acids (FA) and monoacylglycerol (MAG), were respectively 1. 1-, and 1.3-fold less abundant in M145/pWHM3-*sco3201* than in M145/pWHM3. Indeed, FA are likely to be transferred by acyltransferases onto MAG to generate DAG than TAG.

In contrast, membranous phospholipids (PL) such as phosphatidylethanolamine (PE, 1.2-fold), phosphatidylinositol (PI, 1.3-fold), phosphatidylinositolmannosides (PIM, 5.7-fold) and cardiolipin (CL, 1.9-fold) were all less abundant in M145/pWHM3-*sco3201* than in M145/pWHM3 (Figure 1 and Supplementary Figure 2). This indicated that FA generated by the strain are rather being used for TAG than for PL biosynthesis.

### Global Analysis of RNA-Seq Data Revealed a General Repressing Role of SCO3201

**FIGURE 2:**
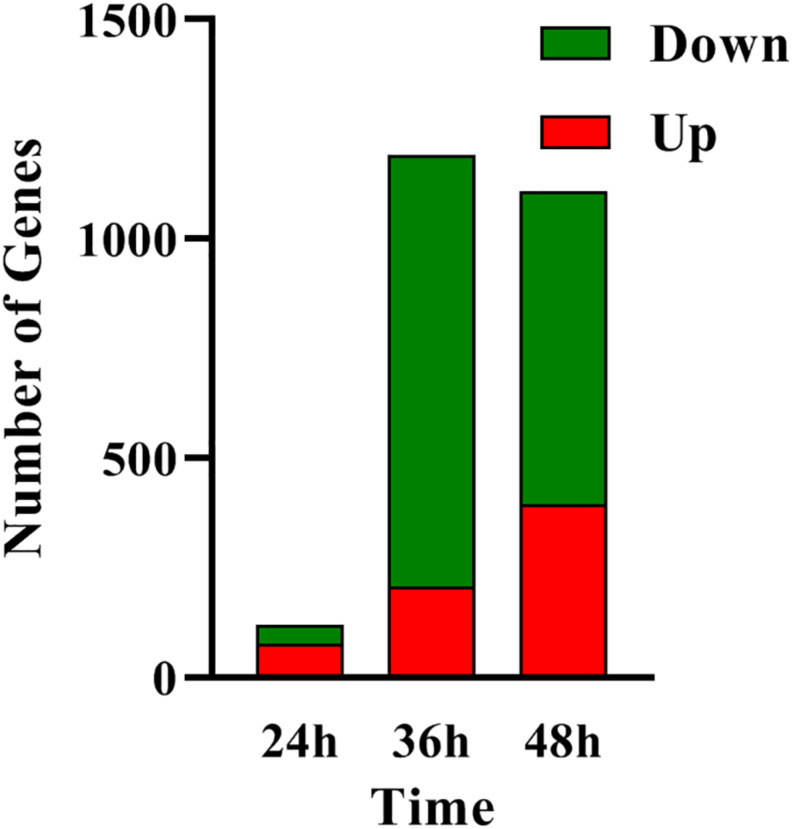
RNA-Seq data: number of genes up- (in red) and down- (in green) regulated in M145/pWHM3-*sco3201* in comparison with M145/pWHM3 at 24, 36, and 48 h, respectively.

In order to determine the molecular processes underpinning the contrasted phenotypic features of the two strains, comparative RNA-sequence analysis (RNA-Seq) of the latter was carried out. Analysis of the data revealed that 121, 1191, and 1108 genes were differentially expressed at 24, 36, and 48 h, respectively, in the two strains. This represents approximately 1.5, 15, and 14% of the genes of *S. coelicolor*, respectively (Figure 2). Eighty three and sixty four percent of the differentially expressed genes were down-regulated at 36 and 48 h, respectively, indicating the preferential repressing role of SCO3201 (Supplementary Data and Figure 2). RNA-Seq data analysis revealed that the transcription of the TetR family regulator SCO3201 itself was 17-fold upregulated in M145/pWHM3-*sco3201*, indicating the successful overproduction of *sco3201* driven by the strong, constitutively expressed *ermE*^∗^ promoter (Supplementary Table 3). RNA-Seq data analysis also revealed the differential expression of numerous genes belonging to various pathways of primary or specialized/secondary metabolism (Table 1 and Supplementary Table 4). Pathways of primary metabolism include starch and sucrose metabolism, glycolysis, fatty acid biosynthesis (FAB) or degradation (FAD), TCA cycle and aromatic amino acids (phe, tyr, trp) biosynthesis or degradation. Detailed comments concerning pathways directing the synthesis and specialized/secondary metabolism are provided in the following section.

**TABLE 1:** RNA-Seq data: List of genes up- or down-regulated in M145/pWHM3-*sco3201* in comparison with M145/pWHM3.

***Gene ID^a^***	**Protein name**	**logFC**	
		**36 h**	**48 h**
**Starch and sucrose metabolism**			
*sco0765*	Secreted endoglucanase	0	–1.092
*sco1879*	Putative secreted pectinesterase	9.994	0
*sco2228*	Alpha-glucosidase	–1.867	–2.593
*sco2649*	Putative 4-alpha-glucanotransferase	–1.955	–1.337
*sco3780*	Putative threalose 6 phosphate hydrolase	–1.297	0
*sco4285*	Putative sugar kinase	–1.308	0
*sco4288*	Putative threalose 6 phosphate phosphatase	–2.402	0
*sco4290*	Putative possible trehalose-phosphate synthase	–1.697	0
*sco5440*	1,4-alpha-glucan branching enzyme	–1.787	–1.534
*sco6110*	Putative glucose kinase/N-acetylmannosamine kinase	–1.625	0
*sco7020*	Secreted alpha-amylase	–1.481	0
*sco7332*	1,4-alpha-glucan branching enzyme	0	–1.325
**Glycolysis**			
*sco2119*	6-phosphofructokinase	0	–1.946
*sco4979*	Putative phosphoenolpyruvate carboxykinase	0	1.559
*sco5679*	Glyceraldehyde 3-phosphate dehydrogenase	–1.370	0
*sco6110*	Putative sugar kinase	–1.625	0
*sco6818*	Phosphoglycerate mutase	1.347	0
*sco7040*	Glyceraldehyde-3-phosphate dehydrogenase	0	1.409
*sco7511*	Glyceraldehyde 3-phosphate dehydrogenase	0	3.915
**Fatty acid biosynthesis**			
*sco0427*	Putative acyltransferase	0	0
***sco0428***	**Putative TetR-family transcriptional regulator**	–1.053	1.789
*sco0548*	Putative 3-oxoacyl-(acyl-carrier-protein) synthase	–3.272	–3.765
*sco1266*	3-oxoacyl-[acyl-carrier-protein] synthase II	0	–2.142
*sco1271*	Putative 3-oxoacyl-[acyl-carrier-protein] synthase III	0	–1.831
*sco1831*	FabG-like 3-ketoacylACP reductase	–1.590	0
*sco2131*	Putative long chain fatty acid CoA ligase	0	1.214
*sco2387*	Malonyl CoA:acyl carrier protein malonyltransferase	0	1.004
*sco3246*	Putative 3-oxoacyl-[acyl carrier protein] synthase III	–1.856	2.109
*sco3248*	Putative 3-oxoacyl-[acyl carrier protein] synthase II	–2.040	2.242
***sco4167***	**Putative TetR-family transcriptional regulator**	–1.601	0
*sco5886*	3-oxoacyl-[acyl-carrier-protein] synthase II	–1.673	–1.702
*sco5888*	3-oxoacyl-ACP synthase III	–1.197	–1.713
*sco6564*	3-oxoacyl-[acyl-carrier-protein] synthase II	0	1.437
**Fatty acid degradation**			
***sco0116***	**Putative TetR-family transcriptional regulator**	1.139	0
***sco0310***	**Putative TetR-family transcriptional regulator**	1.048	0
*sco3247*	Putative acyl-CoA oxidase	–1.568	1.934
*sco4502*	Putative ketoacyl-CoA thiolase	–1.037	0
*sco5399*	Probable acetoacetyl-coA thiolase	1.111	0
*sco6027*	Acetyl-CoA acetyltransferase (thiolase)	1.068	1.342
*sco6475*	FadB-like enzyme	0	–1.366
*sco6787*	Probable acyl-CoA dehydrogenase	0	–1.804
*sco6789*	Putative fatty oxidation protein	0	–1.304
*sco6790*	Putative long chain fatty acid CoA ligase	0	–1.078
***sco6792***	**Putative TetR-family transcriptional regulator**	2.253	
**TCA cycle**			
*sco5831*	Putative citrate synthase-like protein	–1.238	0
*sco4388*	Putative citrate synthase	0	–1.786
*sco1268*	Acyltransferase of pyruvate/2-oxoglutarate dehydrogenase complex,	0	–1.518
*sco1269*	AcoB, pyruvate/2-oxoglutarate/dehydrogenase complex	0	–1.817
*sco1270*	AcoA, pyruvate/2-oxoglutarate/dehydrogenase complex (TPP)	–1.529	–4.042
*sco3831*	bkdA2, branched-chain alpha keto acid dehydrogenase subunit E1	–1.468	0
**Oxidative stress**			
*sco0666*	Catalase (EC 1.11.1.6).	–1.787	0
*sco7590*	Catalase	–1.555	–1.243
*sco6204*	Putative catalase	0	–1.179
**Aromatic amino acids biosynthesis**			
*sco1859*	Putative aminotransferase	–1.243	0
*sco3210*	Putative 2-dehydro-3-deoxyheptonate aldolase	–1.470	1.906
*sco3211*	Putative indoleglycerol phosphate synthase	–1.850	2.150
*sco3212*	Probable anthranilate phosphoribotransferase	–1.892	1.414
*sco3213*	Probable anthranilate synthase component II	–2.142	0
*sco3214*	Probable anthranilate synthase component I	–1.762	1.719
*sco6819*	3-phosphoshikimate 1-carboxyvinyltransferase.	1.425	0
**Aromatic amino acids degradation**			
*sco0199*	Putative alcohol dehydrogenase	0	5.466
*sco1204*	Putative aldehyde dehydrogenase	–1.344	0
*sco1715*	Putative homogentisate 1,2-dioxygenase	–1.336	0
*sco2700*	Tyrosinase (monophenol monooxygenase)	0	–1.967
*sco2927*	Putative 4-hydroxyphenylpyruvate dioxygenase	–1.824	0
*sco3645*	Putative hydrolase	–1.335	0
*sco4681*	Putative short chain dehydrogenase	0	–1.026
*sco7035*	Succinate-semialdehyde dehydrogenase	–1.550	–2.401

*^*a*^The TetR-family genes are in bold. “+”: upregulated in M145/pWHM3-sco3201; “-”: down-regulated in M145/pWHM3-sco3201. “0”: No significant change detected between the two strains on the basis of RNA-Seq data.*

### The Expression of Most Genes of Specialized/Secondary Metabolite Biosynthetic Pathways Was Down-Regulated in *S. coelicolor* Over-Expressing *sco3201*

**FIGURE 3:**
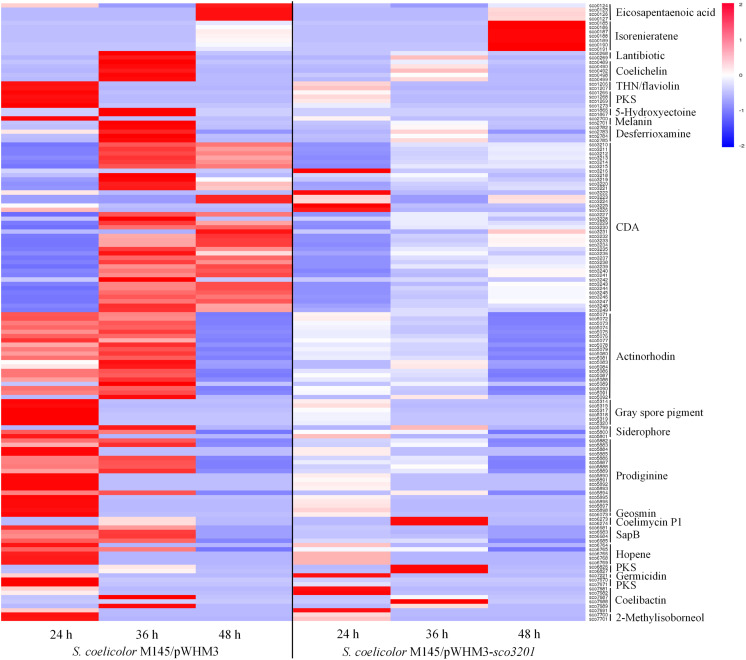
Heat map of transcripts of gene clusters potentially directing the biosynthesis of secondary metabolites with significant abundance change (ANOVA, adjusted *p* < 0.01) between *S*. *coelicolor* M145/pWHM3-*sco3201* and the control strain *S. coelicolor* M145/pWHM3. RNA samples were prepared from cultures of the strains grown on modified R2YE solid medium for 24, 36, and 48 h, respectively. Only transcripts showing significant abundance change between the two strains (ANOVA, adjusted *p* < 0.05) are displayed. Clusters were named according to the nomenclature used previously (Jeong et al., 2016). Gene identifiers are indicated as SCO numbers for both strains. Relative transcript level was indicated on a color scale from red (high) to blue (low) in *S. coelicolor* M145/pWHM3-*sco3201*.

The expression of 23 among the 28 known gene clusters that potentially direct the biosynthesis of secondary metabolites in *S. coelicolor* (Bentley et al., 2002; Challis and Hopwood, 2003) was down-regulated in the strain over-expressing *sco3201* (Figure 3). These included the pathways directing the biosynthesis of CDA (calcium-dependent antibiotic), RED (undecylprodigiosin), ACT (actinorhodin) and gray spore pigment (Figure 3; Feitelson et al., 1985; Malpartida and Hopwood, 1986; Kelemen et al., 1998; Bentley et al., 2002; Hojati et al., 2002).

The expression of 35 among the 39 genes of the CDA cluster (*sco3210*-*sco3249*) was down-regulated at 24 and 36 h (Figure 3 and Supplementary Data), whereas the level of expression of the transcriptional activator CdaR (*sco3217*) (Ryding et al., 2002) remained unchanged at the three time points (Supplementary Data). Interestingly, the expression of the two component system AbsA (*sco3225*)/AbsA2 (*sco3226*) as well as that of *sco3222*, encoding a putative secreted phospholipase A2, and of *sco3216*, encoding a putative cation transport ATPase, was up-regulated in this strain, but only at 24 h (Supplementary Data).

The expression of the genes *sco5882* to *sco5898* of the RED biosynthetic gene cluster (*sco5877*-*sco5898*), was also clearly down-regulated in M145/pWHM3-*sco3201*, whereas the expression of the genes *sco5877* to *sco5881* including the two pathway-specific regulatory genes *redD* (*sco5877*) (Takano et al., 1992) and *redZ* (*sco5881*) (White and Bibb, 1997) remained unchanged (Supplementary Data).

The expression of 19 of the 21 genes of the ACT biosynthetic gene cluster (*sco5071*-*sco5092*) was down-regulated at 36 and 48 h, whereas the expression of the pathway specific TetR-like transcriptional factor ActII-1/AtrA (s*co5082*) as well as that of its regulatory target, ActII-ORF4 (*sco5085*), the activator of the ACT cluster (Uguru et al., 2005), remained unchanged (Supplementary Data).

The expression of other pathways such as those directing the synthesis of eicosapentaenoic acid (*sco0124*-*sco0129*), lantibiotic (*sco0267*-*sco0270*), THN/flaviolin (*sco1205*-*sco1208*), 5-hydroxyectoine (*sco1864*-*sco1867*), melanin (*sco2700*-*sco2701*), gray spore pigment (*sco5314*-*sco5320*), geosmin (*sco6073*), SapB (*sco6681*-*sco6685*), hopene (*sco6764*-s*co6769*), germicidin (*sco7221*), 2-methylisoborneol (*sco7700*-*sco7701*), the siderophores coelichelin (*sco0489*-*sco0499*), desferrioxamine (*sco2782*-*sco2785*), and putative siderophore encoded by *sco5799*-*sco5801* as well as the PKS encoding genes (*sco1265*-*sco1273*) and (*sco7670*-*sco7671*) was also down-regulated in M145/pWHM3-*sco3201* (Figure 3). The transcriptional activity of genes of the coelibactin cluster (*sco7681*-*sco7691*) was rather complex and puzzling, since the expression of *sco7681*-*sco7682* and *sco7691* as well as that of *sco7688* was up-regulated in M145/pWHM3-*sco3201* at 24 and 36 h, respectively, whereas the expression of *sco7687* and *sco7689* was up-regulated in the control strain at 36 h (Figure 3).

In contrast, the expression of the pathways directing the synthesis of the carotenoid pigment, isorenieratene (*sco0185*-*sco0191*), as well as that of the yellow pigment coelimycin P1 (*sco6273*-*sco6274*) and of another polyketide (*sco6826*-*sco6827*) was up-regulated in M145/pWHM3-*sco3201* at 48 and 36 h, respectively (Figure 3). The expression of four genes (*sco7681, sco7682*, *sco7688*, and *sco7691)* belonging to of the coelibactin cluster (*sco7681*-*sco7691*) was also up-regulated in M145/pWHM3-*sco3201* at 24 and 36 h, whereas the expression of two other genes (*sco7687* and *sco7689)* was up-regulated in the control strain at 36 h (Figure 3). This indicated a complex regulation of this pathway.

### The Expression of Most Genes in Fatty Acid Biosynthesis (*fab*) Was Up-Regulated in the *sco3201* Over-Expressing Strain

**FIGURE 4:**
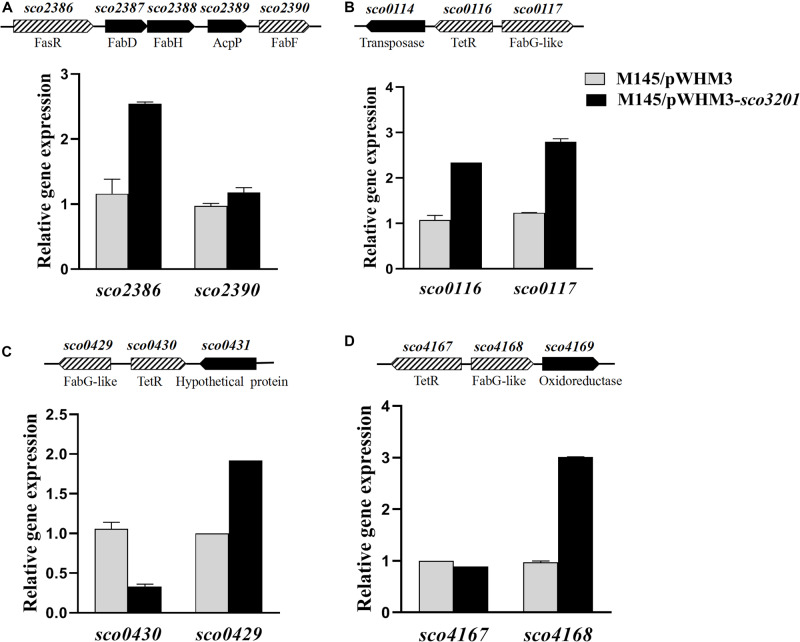
Quantitative analysis of the transcriptional level of *fasR* (*sco2386*) and of putative TetR regulatory targets of SCO3201 (*sco0116*, *sco0430*, *sco4147*) and their neighboring genes by qRT-PCR in the control strain *S. coelicolor* M145/pWHM3 (black histograms) and in *S. coelicolor* M145/pWHM3-*sco3201* (gray histograms) at 72 h. Schematic representation of the genetic surroundings of each TetR regulator encoding gene is provided above each qRT-PCR results. SCO names and putative protein function are shown above and below the genetic maps, respectively. qRT-PCR analysis of the expression level of *sco2386* and *sco2390* **(A)**, *sco0116* and *sco0117* **(B)** *sco0430* and *sco429* **(C)**, and *sco4167* and *sco4168* **(D)**.

Since the lipid content of the control strain and the strain over-expressing s*co3201* greatly varied, the expression of genes involved in fatty acid biosynthesis (*fab*) or degradation (*fad*) was examined in our RNA-Seq data. This revealed that a few *fab* and *fad* genes were differentially expressed in the two strains at 36 and/or 48 h (Table 1 and Figure 4).

The expression of four *fab* genes was up-regulated whereas that of two was down-regulated in M145/pWHM3-*sco3201*. Up-regulated genes include *sco0117* encoding a FabG-like short-chain dehydrogenase/reductase (up-regulated by 2.3-fold at 72 h), *sco2131* encoding a putative long chain fatty acid CoA synthetase (up-regulated by 2.3-fold at 48 h), *sco4168* encoding putative FabG-like protein (up-regulated by 2.4-fold at 72 h) and *sco6564* encoding a FabH2-like 3-oxoacyl-[acyl-carrier-protein] synthase II catalyzing the synthesis of 3-ketoacyl-ACP from malonyl-ACP (Revill et al., 2001) (up-regulated by 2.6-fold at 48 h) (Figure 4 and Table 1). Furthermore, since the regulator FasR is known to be the activator of the major fatty acid biosynthetic operon, *fabDHPF* operon (Arabolaza et al., 2010), we tested its expression level in qRT-PCR and found that it was up-regulated (2.4-fold) in M145/pWHM3-*sco3201* as well as the genes known to be under its positive control, *sco2387* (2-fold) and *sco2390* (1.2-fold) (Figure 4 and Table 1). The up-regulation of these genes is consistent with enhanced fatty acid biosynthesis and thus enhanced TAG content in M145/pWHM3-*sco3201.*

However, in contrast, the expression of some other *fab* genes was down-regulated in M145/pWHM3-*sco3201.* These include *sco0548* encoding a FabB2-like 3-oxoacyl-[acyl-carrier-protein] synthase and *sco1831* encoding a FabG-like 3-ketoacyl ACP reductase that were down-regulated 14- and 3-fold, at 48 and 36 h, respectively (Table 1).

**FIGURE 5:**
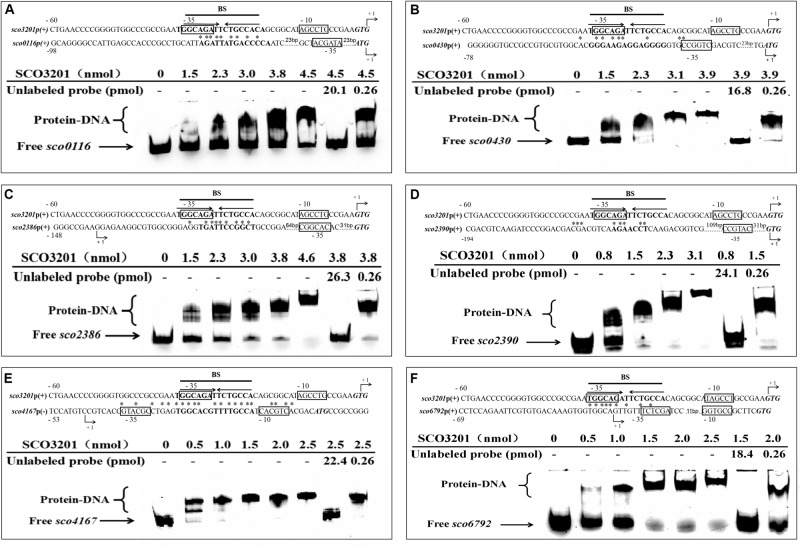
Electrophoretic mobility shift assay of purified SCO3201 with its putative regulatory promoter targets: *sco0116* **(A)**, *sco0430* **(B)**, *sco2386* **(C)**, *sco2390* **(D)**, *sco4167* **(E)**, and *sco6792* **(F)**. In all cases, 0.522 pmol of FITC-labeled probe was used. The specific (unlabeled target promoters) and non-specific (irrelevant DNA) competitors were introduced in lane 7 and lane 8, respectively. BS indicates the core binding site of SCO3201. Putative −10 and −35 regions are boxed. The predicted transcriptional sites (PTS) are indicated by +1 with bent arrows. Translational start codons are in italic and bold. Consensus palindromic sequence of the SCO3201 binding motif is indicated by inverted arrows above the sequence line of the promoter region of *sco3201* on each EMSA result. The nucleotides conserved between the promoter region of a given gene and the BS of SCO3201 are marked by asterisks.

In some of the cases mentioned above, genes encoding TetR regulators were present in the vicinity of *fab* genes/clusters and were also differentially expressed in the two strains as determined by qRT-PCR using RNA prepared from 24, 36, 48, and 72 h grown cultures of both strains. Our data revealed that the up-regulation (by 2.2-fold at 36 h) of the expression of the TetR encoding gene, *sco0116*, in M145/pWHM3-*sco3201*, was correlated with the up-regulation of its divergent gene *sco0117* (Table 1 and Figure 4), whereas, in contrast, the down-regulation (by 0.8-fold at 72 h) of the expression of *sco0430* encoding another TetR regulator, was correlated with the up-regulation (by 1.9-fold at 72 h) of the expression of its divergent gene *sco0429* (Figure 4). Since TetR regulators often regulate the expression of their neighboring genes (Ramos et al., 2005; Cuthbertson and Nodwell, 2013), these observations could suggest that SCO0116 activates the expression of *sco0117* whereas SCO0430 represses that of *sco0429*. Such hypothesis is consistent with the positioning of the putative SCO3201 binding site in the promoter region of these genes, since a conserved BS was found to be located upstream of and in overlap of the promoter regions of *sco0117* and *sco0429*, respectively (Figure 5). In contrast, the up-regulation of the expression of *sco4168* at 72 h was not correlated with any change in the level of expression of its neighboring TetR regulator encoding gene (Figure 4), suggesting that the latter (*sco4167*) does not regulate the expression of *sco4168*. The expression of *sco4168* might thus be regulated directly by SCO3201 or by another TetR regulator under the control of SCO3201.

### The Expression of Most Genes Involved in Fatty Acid Degradation (*fad*) Was Down-Regulated in the *sco3201* Over-Expressing Strain

The expression of 6 *fad* genes (*sco3247*, *sco4502*, *sco6475*, *sco6787*, *sco6789*, and *sco6790*) was down-regulated in M145/pWHM3-*sco3201* whereas the expression of 2 *fad* genes *(sco5399 and sco6027)* was up-regulated (Table 1 and Supplementary Data). *fad* genes whose expression was down-regulated include some genes of the *sco6785*-*sco6790* cluster, encoding proteins putatively involved in fatty acid degradation (FAD), such as *sco6787*/*acdH3* (acyl-CoA-dehydrogenase), *sco6789* (FadB/FadJ-like fatty acid oxidation protein) and *sco6790* (acyl-CoA ligase) (Yang et al., 1988; Menendez-Bravo et al., 2017), whose expression was 3. 5-, 2. 5-, and 2.1-fold down-regulated at 48 h, respectively (Table 1 and Supplementary Figure 2). The down-regulation of the expression of these *fad* genes likely results into a less efficient degradation of fatty acid and thus into a reduced generation of acetyl-CoA and NADPH.

RNA-Seq analysis revealed that the expression of the gene, *sco6792*, encoding the TetR regulator present in the vicinity of this cluster was 4-fold up-regulated in M145/pWHM3-*sco3201* at 24 h (Table 1 and Supplementary Figure 2). This suggests that this regulator represses the expression of genes of this cluster. However, the expression of another gene of this cluster, *sco6788*, encoding a putative thiolase was 2.3-fold up-regulated at 24 h (Table 1 and Supplementary Figure 2) indicating a complex regulation of the expression of some genes of this cluster. Interestingly, the expression of two other thiolases encoding genes, *sco5399* and *sco6027* was also up-regulated (2.1- and 2.5-fold) at 36 and 48 h, respectively (Table 1 and Supplementary Figure 2). These enzymes catalyze the biosynthesis of acyl-CoA from acyl chain and CoA or conversely the release of CoA from acyl-CoA. They are therefore involved in both biosynthetic and degradative pathways and their expression might thus be subjected to a specific regulation.

However, in some cases the putative regulatory interaction between a TetR regulator and its neighboring genes involved in lipid metabolism could not even be hypothesized. For instance, the expression of the TetR regulator encoding gene, *sco0310*, was 2-fold up-regulated in M145/pWHM3-*sco3201* (Table 1 and Supplementary Figure 2), whereas the level of expression of its neighboring genes, *sco0311* encoding a FadD-like long-chain-fatty-acid-CoA ligase and *sco0312* encoding a FadE-like acyl-CoA dehydrogenase (Menendez-Bravo et al., 2017), was unchanged. Similarly, the expression of *sco6475*, encoding a FadB-like 3-hydroxyacyl-CoA dehydrogenase, was shown to be 2.6-fold down-regulated in M145/pWHM3-*sco3201* at 48 h (Table 1 and Supplementary Figure 2), whereas the expression of its close-by TetR regulator encoding gene, *sco6474*, remained unchanged. This suggested that the expression of these genes might be controlled by other regulators under the control of SCO3201, since no SCO3201 binding site (BS) was detected in their promoter region.

### Identification of SCO3201 Binding Sites in the Promoter Regions of Its Putative Regulatory Targets

In order to establish the ability of SCO3201 to interact with the promoter region of its putative targets genes defined above, the MEME program^1^, was used to search the previously identified palindromic binding site of SCO3201 (BS: TGGCAGATTCTGCCA) (Xu et al., 2010) in the promoter regions of these genes. Putative operator sequences bearing similarity to SCO3201 binding site were discovered in the promoter regions of the TetR regulators encoding genes *sco0116*, *sco0430*, *sco4167*, and *sco6792* as well as in that of *fasR* (*sco2386*), encoding the positive regulator of the expression of the major fatty acid biosynthetic operon, *fabD-H-acp-fabF operon* and of *fabF* (*sco2390*), a member of this operon (Figure 5; Arabolaza et al., 2010).

EMSA was performed using FITC-labeled target promoter regions and His_6_-SCO3201 purified from *E. coli*. The results indicated that purified His_6_-SCO3201 was able to specifically bind and delay the migration of the fragments carrying the promoter regions of these target genes (Figure 5). A competition assay was performed using excess amounts of unlabeled target promoter region to the reaction mixture. This successfully out-competed the specific interactions and eliminated the presence of the delayed DNA fragments (Figure 5, lane 7). In contrast, when another DNA fragment (+11 to +444 relative to the translational start site of *sco3201*) was added, the delayed migration of the shifted bands remained, indicating the absence of competition (Figure 5, lane 8). Altogether, these data demonstrated the specificity of the interaction of SCO3201 with the promoter regions of these genes. The putative SCO3201 binding sites in the promoter regions of *sco0430* and *sco4167* overlapped their −35 promoter regions (Figure 5). The location of this BS was predicted to impair RNA polymerase binding leading to down-regulation of the expression of these genes. This prediction was verified by qRT-PCR experiment for *sco0430* but not for *sco4167* whose expression was similar in the two strains (Figure 4). Our EMSA results also demonstrated direct binding of SCO3201 to the promoters regions of *sco2386* (activator FasR) and *sco2390* (FabF-like), and the position of the BS of SCO3201, upstream of the −35 region (position of activator site), is consistent with the activation of the expression of these genes by SCO3201 as shown in Figure 4.

Our EMSA results thus demonstrated the specific binding of His_6_-SCO3201 to the target promoter regions and most of them are consistent with RNA-Seq and qRT-PCR data. This indicated that, *in vivo* and in conditions of over-expression, SCO3201 likely regulates positively or negatively the expression of these genes.

## Discussion

**FIGURE 6:**
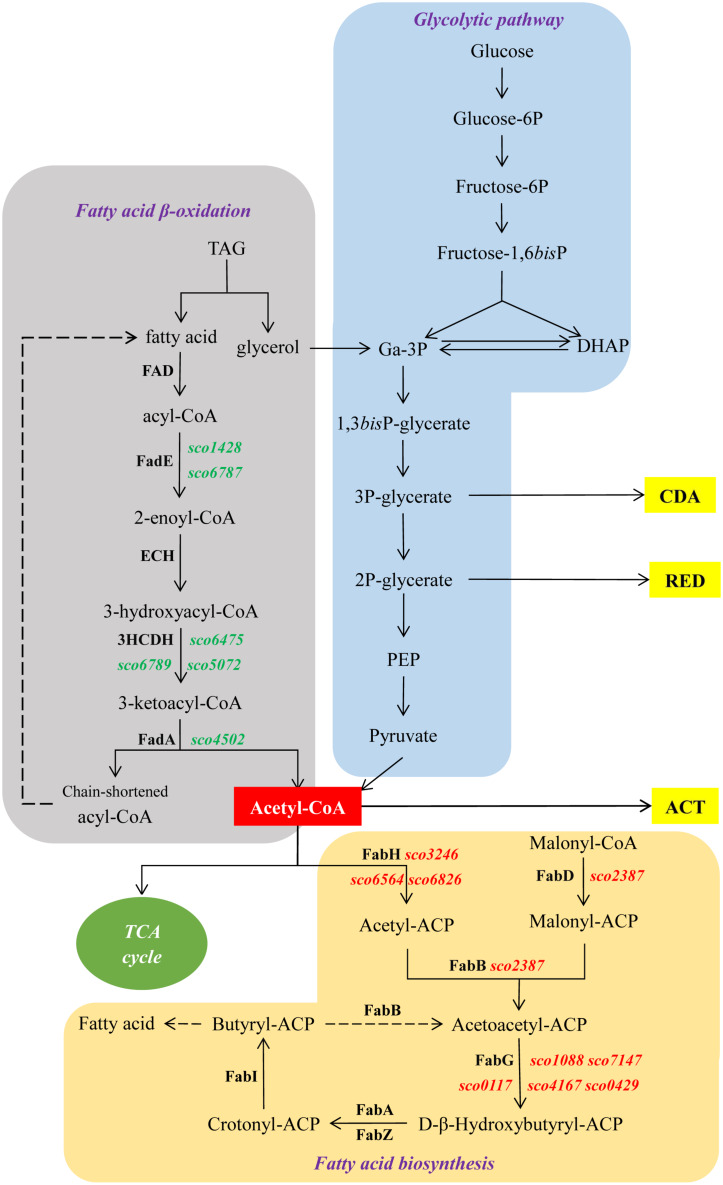
Schematic representation of central metabolic pathways of *S. coelicolor*. In green and red, genes up- and down-regulated in M145/pWHM3-*sco3201*, respectively. Dashed arrows indicate multiple reactions.

The over-expression of *sco3201*, encoding a TetR regulator, was previously shown to inhibit antibiotic production and morphological differentiation in *S. coelicolor* (Xu et al., 2010). In this study, we demonstrated that these phenotypic changes were correlated with higher TAG content and lower phospholipid (PL) content as well as with the up-regulation of the *fab* genes and down-regulation of the *fad* genes found to be differentially expressed in the two strains (Table 1 and Figures 4, 6). This regulatory impact could be either direct or indirect, mediated by other TetR regulators present or not in the vicinity of *fab* or *fad* genes/clusters.

The enhanced TAG synthesis that requires enhanced fatty acid (FA) synthesis combined with predicted reduced FA degradation linked to down-regulation of the expression of *fad* genes is expected to lead to a reduction of acetyl-CoA availability in M145/pWHM3-*sco3201.* The enhanced TAG content and thus enhanced TAG biosynthesis in M145/pWHM3-*sco3201* might be responsible for the lower PL content since the biosynthesis of these two lipids classes use the same FA pool. Furthermore, PL being major phosphorus reservoirs, reduced PL synthesis is expected to lead to phosphorus saving leading to higher internal phosphate availability. Consequently, reduced availability of acetyl-CoA due to enhanced TAG synthesis and putative enhanced availability of internal phosphorus due to reduced PL synthesis might both contribute to the inhibition of the biosynthesis of the canonical antibiotics usually produced by this strain.

However, since other specialized metabolites requiring acetyl-CoA as precursors are being produced by the *sco3201* over-expressing strain, the poor acetyl-CoA availability might not be the main cause of the absence of biosynthesis of these antibiotics. The main cause might rather be related to the absence, in the *sco3201* over-expression strain, of the specific physiological signals involved in the triggering of the expression of the cognate biosynthetic pathways. The constitutive oxidative metabolism, characteristic of *S. coelicolor* and that of the *ppk* mutant of *S. lividans* mainly seen in condition of phosphate limitation, is linked to energetic stress (Esnault et al., 2017; Aaron et al., 2020; Virolle, 2020). Indeed, genes of the Pho regulon involved in phosphate supply were shown to be poorly expressed in *S. coelicolor* (Aaron et al., 2020) and the *ppk* mutant of *S. lividans* lacks a polyphosphate kinase, an important enzyme involved in the regeneration of ATP from ADP and polyphosphate (Chouayekh and Virolle, 2002; Ghorbel et al., 2006). In order to re-establish their energetic balance, these two strains activate, at least temporarily, their oxidative metabolism (Esnault et al., 2017). Acetyl-CoA is thus used to feed the TCA cycle and cannot be stored as TAG. An oxidative metabolism is known to generate ROS and NOS and the latter were proposed to play a role in the induction of antibiotic biosynthesis in this strain (Esnault et al., 2017; Virolle, 2020) as in other *Streptomyces* (Beites et al., 2011, 2015; Miranda et al., 2014; Prajapati et al., 2019). These bio-active molecules were proposed to fulfill specific functions in the producing bacteria (Esnault et al., 2017; Virolle, 2020). For instance, CDA and RED were proposed to create damage to the membrane contributing to cell death and lysis of a fraction of the population (Esnault et al., 2017; Tenconi et al., 2018; Virolle, 2020). This would provide nutriments, and especially phosphate, to support the activation of the oxidative metabolism of the surviving population. Furthermore, since the onset of ACT biosynthesis was shown to coincide with an abrupt drop in the intracellular ATP concentration in *S. coelicolor*, ACT, as other molecules possessing quinone groups (melanine, humic acid …), was proposed to act as electron acceptor (Esnault et al., 2017). ACT would capture electrons of the respiratory chain and doing so would reduce oxidative stress as well as respiration efficiency to reduce ATP generation in order to adjust it to phosphate scarcity (Esnault et al., 2017; Virolle, 2020).

We propose that the activation of the oxidative metabolism that takes place in the native and control strains of *S. coelicolor* does not occur in the strain over-expressing *sco3201*. In that strain, acetyl-CoA availability is probably low since it is stored as TAG and its generation *via* FA degradation is down-regulated. This would impair sufficient feeding of the TCA cycle to support an active oxidative metabolism. Furthermore, a putatively higher Pi availability due to reduced PL synthesis, might contribute to repress antibiotic biosynthesis in *Streptomyces* (Martin and Liras, 2012). This might be due to the fact that the activation of the oxidative metabolism does not take place in conditions of phosphate abundance, as demonstrated in the *ppk* mutant of *S. lividans* (Chouayekh and Virolle, 2002). Low acetyl-CoA availability and possibly also enhanced Pi availability are proposed to impede the activation of the oxidative metabolism of this strain. In consequence, the signals triggering the expression of the antibiotic biosynthetic pathways are not generated in this strain and the expression of these pathways is not activated. The hypothesis of the poor activation of the oxidative metabolism of this strain is supported by the fact that the expression of most genes of the TCA cycle was down-regulated in M145/pWHM3-*sco3201*, at least at 36 h (Table 1). In consequence, TCA might not provide sufficient precursors for amino acids biosynthesis and the expression of genes involved in amino acids metabolism as well as that of genes belonging to the clusters directing the biosynthesis of the antibiotics CDA and RED, was also down-regulated (Figure 3).

In contrast, genes of the biosynthetic pathways directing the synthesis of the carotenoid pigment, isorenieratene, the yellow pigment coelimycin P1 and of another polyketide encoded by the genes *sco6826*-*sco6827* were highly expressed in the *sco3201* over-expressing strain (Figure 3). The expression of these pathways is likely to be related to the specific physiological state of this strain. We propose that the strain over-expressing *sco3201* is depleted in NADPH since active fatty acid and thus TAG biosynthesis consumes NADPH and the latter is not regenerated by fatty acid degradation since *fad* genes expression was shown to be down-regulated in this strain. NADPH is a crucial co-factor for the activity of thioredoxins, enzymes involved in the reduction of non-native disulfide bonds formed in proteins in conditions of oxidative stress (Lu and Holmgren, 2014). Its consumption for other purpose might lead to the raise of a specific oxidative stress that might be responsible for the onset of the biosynthesis of isorenieratene, a carotenoid pigment known to possess anti-oxidant function (Galasso et al., 2017; Chen et al., 2019). The concomitant strong expression of the CPK cluster directing the synthesis of coelimycin P1 (Bednarz et al., 2019) and of another polyketide (*sco6826*-*sco6827*) in the *sco3201* over-expressing strain suggests that these molecules might have similar anti-oxidant function.

At last, it is noteworthy that whereas the expression of most biosynthetic genes of the CDA, RED, and ACT clusters was lower in M145/pWHM3-*sco3201* than in M145/pWHM3, the expression of their pathway-specific transcriptional activators (encoded by *cdaR*, *redD*, *redZ*, and *actII-ORF4*) remained at a similar level in both strains (Figure 3). This suggested that, these regulators are present but unable to fulfill their regulatory function in M145/pWHM3-*sco3201*. To be functional, these cluster-situlated regulators (CSR) require the binding of specific ligands that would be depleted in the *sco3201* over-expressing strain. Several reports in the literature mention that biosynthetic intermediates or even end-products of biosynthetic pathways interact with CSR and auto-control either positively, if poorly abundant, or negatively, if highly abundant, the expression of the corresponding pathway (Kong et al., 2019). If the expression level of the biosynthetic pathways is null in the strain over-expressing *sco3201*, positive regulation by intermediates or end products of the pathway cannot take place. Furthermore, gamma-butyrolactone synthetized by ScbA was shown to play a positive role in the regulation of the expression of the RED and ACT clusters (Takano et al., 2001; D’Alia et al., 2011) and our previous work indicated that SCO3201 represses *scbA* expression at least at early time points (Xu et al., 2010). A reduced butyrolactone synthesis in the *sco3201* over-expressing strain may thus also account for low ability of this strain to produce these antibiotics.

## Conclusion

Our study contributed to elucidate the molecular processes underlying the inhibition of production of the canonical antibiotics usually produced by *S. coelicolor* in the strain over-expressing the TetR-regulator SCO3201. The absence of synthesis of these molecules was clearly correlated with an enhanced TAG content and a reduced PL content of this strain. This resulted into reduced acetyl-CoA availability as well as possibly enhanced internal phosphate availability that would preclude the activation of the oxidative metabolism of this strain that is thought to generate signaling molecules triggering the biosynthesis of the canonical antibiotics. Altogether, our results thus suggest that impaired TAG biosynthesis or enhanced TAG degradation would enhance acetyl-CoA availability and thus improve the feeding of the TCA cycle to support an active oxidative metabolism. Such strategies are promising to enhance antibiotic biosynthesis in *Streptomyce*s species as already demonstrated by several published studies (Banchio and Gramajo, 2002; Foley et al., 2009; Craney et al., 2012; Chen et al., 2020).

## Data Availability Statement

The datasets presented in this study can be found in online repositories. The names of the repository/repositories and accession number(s) can be found in the article/ Supplementary Material.

## Author Contributions

JZ contributed to experimental materials and reagents, execution of the experiments, and data management and reporting. QL, SA, and CL were involved in managing experimental materials and reagents and execution of the experiments. ZX executed the experiments. MC helped with data management and interpretation and literature review. QZ provided personnel, environmental and financial support, tools and instruments that were vital for the project, and constructed idea or hypothesis for research and manuscript. MD executed the experiments, managed and interpreted the data. PC contributed to data management and interpretation, reviewed the manuscript before submission for both grammar and intellectual content. M-JV supervised the course of the project or article and reviewed the manuscript before submission. DX constructed idea and hypothesis for research, logical interpretation of the results, and constructed and drafted the whole manuscript. All authors contributed to the article and approved the submitted version.

## Conflict of Interest

The authors declare that the research was conducted in the absence of any commercial or financial relationships that could be construed as a potential conflict of interest.

## Footnotes

1 http://meme-suite.org/
