# Supplementary material for: The Inhibition of Antibiotic Production in Streptomyces coelicolor Over-Expressing the TetR Regulator SCO3201 IS Correlated With Changes in the Lipidome of the Strain
Source: Front Microbiol. 2020 Jun 23;11:1399. doi: 10.3389/fmicb.2020.01399 (PMC7324645; doi:10.3389/fmicb.2020.01399)
Supplement: Supplementary file 2 [file Data_Sheet_2.docx]

**Supplementary Table 1 Synthetic oligonucleotides used in this study.**

| Primer | 5’ → 3’ sequence^a^ | Positions | Purpose |
| --- | --- | --- | --- |
| ExpTetR1-NdeI | CAA**CATATG**GTGAGCAGCACCATTCCAGCACTTC | +1 to +708 | Amplification of *sco3201* for protein expression |
| ExpTetR2-XhoI | CAA**CTCGAG**CCCCTCTTCCGCGGGCCC |  |  |
| 0116F | AGCCAGTGGCGATAAGCGCCGTACACCTTGG | -219 to +43 | Amplification of the *sco0116* promoter for EMSA |
| 0116R | AGCCAGTGGCGATAAGGCCTGCGGTTTTCCT |  |  |
| 0430F | AGCCAGTGGCGATAAGTAGAACTGGGCGGTGT | -238 to +32 | Amplification of the *sco0430* promoter for EMSA |
| 0430R | AGCCAGTGGCGATAAGTCGTAAAGCGGAGGGT |  |  |
| 2386F | AGCCAGTGGCGATAAGACCCAGGTGACGA | -268 to +1 | Amplification of the *sco2386* promoter for EMSA |
| 2386R | AGCCAGTGGCGATAAGCGGGGACAAGACT |  |  |
| 2390F | AGCCAGTGGCGATAAGTCCTTCACCGAC | -265 to -7 | Amplification of the *sco2390* promoter for EMSA |
| 2390R | AGCCAGTGGCGATAAGCTTTCTCCAACG |  |  |
| 4167F | AGCCAGTGGCGATAAGGAGCCGTTCCG | -234 to +19 | Amplification of the *sco4167* promoter for EMSA |
| 4167R | AGCCAGTGGCGATAAGTGCGACCACCC |  |  |
| 6792F | AGCCAGTGGCGATAAGGGCAGGCACA | -153 to +100 | Amplification of the *sco6792* promoter for EMSA |
| 6792R | AGCCAGTGGCGATAAGGAAGCGGCAC |  |  |
| Plabel | AGCCAGTGGCGATAAG |  | FITC labeling |
| RT-0116F | CTCGCCACCGACATCG | +337 to +438 | RT-PCR of *sco0116* |
| RT-0116R | GTCACGGGCCAGGAACT |  |  |
| RT-0117F | CTGCTCGGAGGCGACATGA | +253 to +397 | RT-PCR of *sco0117* |
| RT-0117R | ACCAGGACAGGGCGGAAAG |  |  |
| RT-0310F | CGAGACGGGCTGGG | +630 to +830 | RT-PCR of *sco0310* |
| RT-0310R | ATCTTCACGGGGAGGTT |  |  |
| RT-0312F | TGGCGGACAGCGAG | +557 to +781 | RT-PCR of *sco0312* |
| RT-0312R | GGGAGGGGCAGTGG |  |  |
| RT-0429F | AGTGATCCGTGCTGCGCT | +237 to +389 | RT-PCR of *sco0429* |
| RT-0429R | CCGAGGCTCCTGAGGTTGTC |  |  |
| RT-0430F | TACTCAACACCCTCCACAG | +436 to +602 | RT-PCR of sco0430 |
| RT-0430R | CACGCCCAGCAGCAT |  |  |
| RT-0548F | ACGGCTTCGTGATGGGG | +722 to +955 | RT-PCR of *sco0548* |
| RT-0548R | TGGCGTGGGCGTTGAC |  |  |
| RT-1831F | CGGACGAGCAGTGGCAGT | +302 to +466 | RT-PCR of *sco1831* |
| RT-1831R | GGCGCAGACCGTTGGA |  |  |
| RT-2131F | CGGCATCGGCTTCACG | +1092 to +1283 | RT-PCR of *sco2131* |
| RT-2131R | GGGTTGTTCCAGTACTCCTTGA |  |  |
| RT-2386F | ATCGCCTCCGACCCCT | +933 to +1046 | RT-PCR of *sco2386* |
| RT-2386R | GCTCGCCTGTTCGAGATAGAC |  |  |
| RT-2387F | CCACACCCGCCACA | +600 to +804 | RT-PCR of *sco2387* |
| RT-2387R | GCCCAGCTCCTTGAAC |  |  |
| RT-2390F | CCAAGAACAACGACGACCC | +665 to +909 | RT-PCR of *sco2390* |
| RT-2390R | GTTGCCGTCCAGCAGGTT |  |  |
| RT-4167F | CGACGCTGACCGAACG | +59 to +223 | RT-PCR of *sco4167* |
| RT-4167R | CCTGCTTGGAGCGGAA |  |  |
| RT-4168F | CGGGTCCTCACGGTGAAC | +367 to +588 | RT-PCR of *sco4168* |
| RT-4168R | GGCGGTGAAGCGGATG |  |  |
| RT-5399F | CCGCCCTGTCCCAC | +554 to +802 | RT-PCR of *sco5399* |
| RT-5399R | GGCTCATCACGACCACG |  |  |
| RT-6027F | CGAGACGGGCTGGG | +630 to +830 | RT-PCR of *sco6027* |
| RT-6027R | ATCTTCACGGGGAGGTT |  |  |
| RT-6474F | CTGTTCACCCGCTATCTG | +454 to +667 | RT-PCR of *sco6474* |
| RT-6474R | TGCCCGTGCCGAAG |  |  |
| RT-6475F | CGGCTTCTACACCTACGA | +861 to +1020 | RT-PCR of *sco6475* |
| RT-6475R | CTTGGCGAACACCTCC |  |  |
| RT-6564F | TACCGCTGGGCCACCA | +631 to +856 | RT-PCR of *sco6564* |
| RT-6564R | CCCGCTCCGCCAACTT |  |  |
| RT-6785F | ACCTCCTCGACGGAGGCT | +689 to +877 | RT-PCR of *sco6785* |
| RT-6785R | GTGCGGTGAACAGGGCTT |  |  |
| RT-6786F | TCGGCAAACCCCTCAAGC | +443 to +583 | RT-PCR of *sco6786* |
| RT-6786R | CGTGTTCCGGCGCGTA |  |  |
| RT-6787F | CGTCCGCTTCGAGATCGC | +849 to +1028 | RT-PCR of *sco6787* |
| RT-6787R | CCGCCGTGGAGTTGCA |  |  |
| RT-6788F | CCGCCGAGAGCCTG | +644 to +832 | RT-PCR of *sco6788* |
| RT-6788R | GGTCACCGACCTCCTTG |  |  |
| RT-6789F | GGTATCGCCTACTCGTGTG | +1009 to +1152 | RT-PCR of *sco6789* |
| RT-6789R | CGCCTTCTCCTGAATCGT |  |  |
| RT-6790F | CGGATTTGGGAGAAGC | +853 to +1045 | RT-PCR of *sco6790* |
| RT-6790R | GCAGCGGCAACAGG |  |  |
| RT-6792F | AGGGGTTCGTGGTCGC | +283 to +527 | RT-PCR of *sco6792* |
| RT-6792R | CGGGTTGAGCAGGAAGGAG |  |  |

^a^Engineered restriction enzyme sites are in bold. Underlined nucleotides show no homology to the template; they were used for FITC labeling.

**Supplementary Table 2. Quaternary gradient mobile phase composition. A, isooctane:ethyl acetate (99.8:0.2, v/v); B, acetone:ethyl acetate (2:1, v/v) containing 0.15% acetic acid (v/v); C, 2-propanol:water (85:15, v/v) containing 0.043% acetic acid (v/v) and 0.104% triethylamine (v/v); D, ethyl acetate.**

| **Time (min)** | **Percent solvent** | |  |  | **Flow-rate (mL/min)** |
| --- | --- | --- | --- | --- | --- |
|  | **A** | **B** | **C** | **D** |  |
| **0** | **100** | **0** | **0** | **0** | **0.8** |
| **1.5** | **100** | **0** | **0** | **0** | **0.8** |
| **1.6** | **97** | **3** | **0** | **0** | **0.8** |
| **9** | **94** | **6** | **0** | **0** | **0.8** |
| **11** | **70** | **30** | **0** | **0** | **0.8** |
| **14** | **45** | **55** | **0** | **0** | **0.8** |
| **15** | **45** | **55** | **0** | **0** | **0.8** |
| **16** | **40** | **55** | **5** | **0** | **0.8** |
| **20** | **35** | **55** | **10** | **0** | **0.8** |
| **20.1** | **33** | **50** | **17** | **0** | **0.8** |
| **25** | **38** | **45** | **17** | **0** | **0.8** |
| **25.1** | **48** | **35** | **17** | **0** | **0.8** |
| **30** | **53** | **30** | **17** | **0** | **0.8** |
| **40** | **40** | **0** | **60** | **0** | **0.8** |
| **40.1** | **0** | **100** | **0** | **0** | **0.8** |
| **42** | **0** | **100** | **0** | **0** | **0.8** |
| **42.1** | **50** | **0** | **0** | **50** | **0.8** |
| **45** | **50** | **0** | **0** | **50** | **0.8** |
| **47** | **100** | **0** | **0** | **0** | **0.8** |
| **53** | **100** | **0** | **0** | **0** | **0.8** |

**Supplementary Table 3. RNA seq data of differentially expressed TetR-family genes at three tested time points in the two strains. The strain M145/pWHM3 being taken as reference.**

| TetR genes^a`^ | logFC | | |
| --- | --- | --- | --- |
|  | 24h | 36h | 48h |
| ***sco0116*** | 0 | 1.139 | 0 |
| ***sco0310*** | 0 | 1.048 | 0 |
| ***sco0428*** | 0 | -1.052 | 1.788 |
| *sco2223* | 0 | 0 | 1.136 |
| *sco2994* | 0 | 0 | 1.070 |
| *sco3201* | 1.664 | 3.034 | 4.081 |
| *sco3207* | 0 | 1.049 | 0 |
| ***sco4167*** | 0 | -1.600 | 0 |
| ***sco6792*** | 2.253 | 0 | 0 |
| *sco5209* | 0 | -1.261 | 0 |
| *sco7539* | 0 | 0 | 1.238 |

^a^The TetR-family genes adjacently located to the fatty acid metabolism gene cluster are in bold.

**Supplementary Table 4. The most enriched metabolic pathways at three tested time points.**

| 24 h | 36 h | 48 h |
| --- | --- | --- |
| Phenylalanine, tyrosine and tryptophan biosynthesis | Phenylalanine, tyrosine and tryptophan biosynthesis | Fatty acid biosynthesis |
| Fatty acid metabolism | Tyrosine metabolism | alpha-Linolenic acid metabolism |
| alpha-Linolenic acid metabolism | Tryptophan metabolism | Sphingolipid metabolism |
| Synthesis and degradation of ketone bodies | Fatty acid biosynthesis | Glycosphingolipid biosynthesis - globo series |
| Biosynthesis of unsaturated fatty acids | Alanine, aspartate and glutamate metabolism | beta-Alanine metabolism |
| Lysine degradation | Glyoxylate and dicarboxylate metabolism | Galactose metabolism |
| Inositol phosphate metabolism | Starch and sucrose metabolism | Inositol phosphate metabolism |
|  | Butanoate metabolism | Other glycan degradation |
|  | Porphyrin and chlorophyll metabolism |  |
|  | Nitrogen metabolism |  |


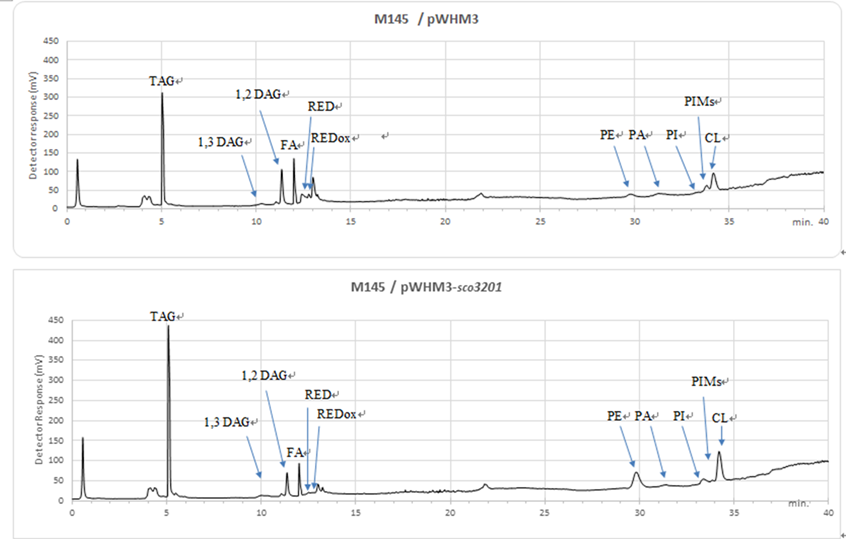


**Supplementary Fig. 1. Results of LC-Corona.**


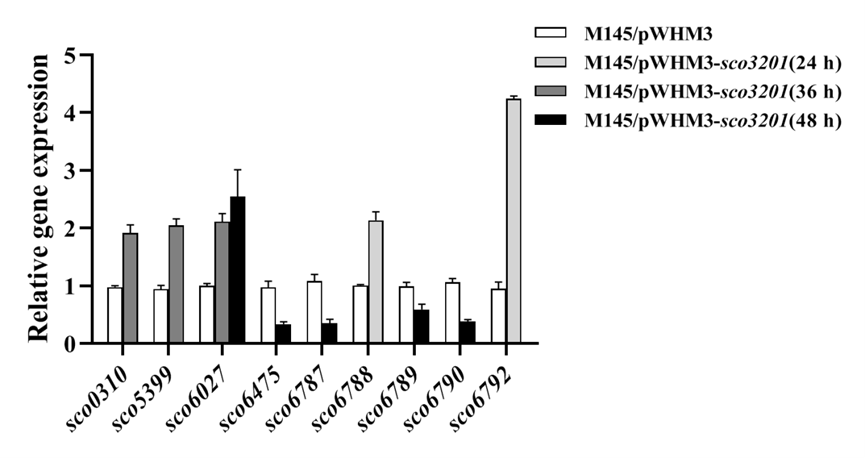


**Supplementary Fig. 2. qRT-PCR analysis of the expression level of various FAD associated genes.**
